# Affected Others Responsivity to Gambling Harm: An International Taxonomy of Consumer-Derived Behaviour Change Techniques

**DOI:** 10.3390/jcm10040583

**Published:** 2021-02-04

**Authors:** Natalia Booth, Nicki A. Dowling, Jason Landon, Dan I. Lubman, Stephanie S. Merkouris, Simone N. Rodda

**Affiliations:** 1 School of Population Health, University of Auckland, Private Bag 92019, Auckland 1142, New Zealand; natalia.booth@auckland.ac.nz; 2 School of Psychology, Deakin University, Geelong, VIC 3220, Australia; nicki.dowling@deakin.edu.au (N.A.D.); stephanie.merkouris@deakin.edu.au (S.S.M.); 3 Department of Psychology and Neuroscience, Auckland University of Technology, Auckland 1010, New Zealand; jason.landon@aut.ac.nz; 4 Eastern Health Clinical School and Monash Addiction Research Centre, Monash University, 5 Arnold St, Box Hill, VIC 3800, Australia; dan.lubman@monash.edu; 5 Turning Point, Eastern Health, 110 Church St, Richmond, VIC 3121, Australia

**Keywords:** self-help, concerned significant others, treatment, behaviour change, behaviour change techniques, gambling harm

## Abstract

Affected others impacted by someone else’s gambling utilise numerous behaviour change strategies to minimise gambling-related harm but knowledge on what these strategies are and how they are implemented is limited. This study aimed to develop a comprehensive data-driven taxonomy of the types of self-help strategies used by affected others, and to categorize these into high-level behaviour change techniques (BCTs). Two taxonomies were developed using an inductive and deductive approach which was applied to a dataset of online sources and organised into the Rubicon model of action phases. These taxonomies were family-focused (how to reduce the impact of gambling harm on families) and gambler-focused (how to support the gambler in behaviour change). In total, 329 online sources containing 3536 different strategies were identified. The family-focused classification contained 16 BCTs, and the most frequent were professional support, financial management and planned consequences. The gambler-focused classification contained 11 BCTs, and the most frequent were feedback on behaviours, professional support and financial management. The majority of family- and gambler-focused BCTs fell under the actional phase of the Rubicon model. Grounded in lived experience, the findings highlight the need for intervention and resource development that includes a wide range of specific techniques that affected others can utilise.

## 1. Introduction

Gambling Disorder is classified in the fifth edition of the Diagnostic and Statistical Manual as a behavioural addiction alongside substance use disorders [1]. It is characterized by a pattern of persistent and recurrent gambling that is associated with substantial impairment or distress [1]. Evidence suggests that the most efficacious treatment for Gambling Disorder is cognitive-behavioural therapy with weaker evidence for pharmacotherapy, self-help and motivational interviewing [2]. The negative impacts of having a gambling problem range from financial harm, emotional or psychological distress as well as relationship disruption and conflict [3].

Gambling-related harm can be experienced not only by the person with the gambling problem but their family, friends, colleagues, and the wider community [4,5,6]. Recent studies indicate that for every person with a gambling problem, there are approximately six others affected [7]. Moreover, international estimates suggest that between 2% and 19% of individuals may be impacted by someone’s gambling problem [8,9,10,11,12,13,14]. Harm experienced by affected others (inclusive of partners, children, parents, grandparents and siblings, as well as close friends and work colleagues) includes financial harm; relationship disruption, conflict or breakdown; emotional and psychological distress; decrements to health; cultural harm; reduced performance at work or study; criminal activity; and harm to life course and intergenerational harm [3,6,15]. It is estimated that affected others may carry 1.5-fold more gambling harm than people who gamble [6]. Most of the burden of harm falls on the partners of people with gambling problems [4,5].

Treatment approaches for affected others involve family-focused and gambler-focused approaches [16]. Family-focused approaches aim to reduce the impact of gambling harm on families with or without the involvement of the gambler. There are a range of family-focused treatments, including the 5-Step Method and Coping Skills Training (CST). The 5-Step Method is based on the Stress–Strain–Coping–Support (SSCS) model which argues that the chronic stress of having a gambling problem in the family results in strain on affected others, causing a departure from a state of well-being. According to the SSCS model, coping strategies and social support play an important role in the stress–strain relationship [17]. The content of the 5-Step Method includes (1) supporting affected others to explore concerns and needs, (2) providing relevant information, (3) exploring coping responses, (4) helping to create a network of social support, and (5) discussing future needs including specialist help [18,19]. Similarly, CST is based on the family stress and coping model, which postulates that the distress experienced by the affected other is a result of the gambler’s behaviour in combination with a reduced ability to cope with the gambling [20,21,22]. Hence, CST aims to help affected others cope with this distress [20,23]. Other family-focused treatments also include training on how to respond to relapse, communication skills, financial management, and problem solving [24].

Gambler-focused treatment approaches involve affected others with the purpose of supporting the gambler in behaviour change [16,25,26,27,28,29,30]. Gambler-focused approaches aim to provide education, advice and counselling for affected others and are a primary reason family contact help services [16,31]. Many treatments, such as Community Reinforcement and Family Training (CRAFT), have a blended approach which focuses on improving individual functioning and the quality of the relationship, as well as training in communication, stress reduction, problem solving and financial management and learning skills to encourage gambling reduction and help seeking [27,29,30,32,33].

A recent systematic review identified seven treatment studies for affected others [34]. Three of these studies were family focused [19,20,24] and four offered a blended approach that included both family- and gambler-focused strategies [27,29,30,33]. The meta-analysis conducted by Merkouris, Dowling and Rodda [34] indicated that face-to-face therapist-delivered psychosocial treatment was associated with improved depressive symptomatology, affected other coping, treatment entry for the person with addiction and marital/relationship discord compared to control groups. In terms of self-directed treatments, all studies that delivered CRAFT-based interventions were associated with no significant differences between treatments and control groups. Due to the small number of studies, however, these findings need to be interpreted with caution.

Over recent years, there has been growing recognition that most affected others do not engage with professional treatments and instead use a range of self-management or self-help approaches to mitigate harm [15,31,35]. Rodda, Dowling, Thomas, Bagot and Lubman [16] reported that 90% of affected others seeking help online reported use of behaviour change strategies. This included talking about gambling to other affected others (78%), reading information about gambling (70%), trying different strategies to reduce harm such as budgeting or avoidance (63%), and using online gambling forums (40%). Côté et al. [36] conducted the first study to examine these self-help strategies in detail. In interviews with nine partners of gamblers (8 female), they identified 18 gambler-focused strategies that were used to influence the gambler’s behaviour. These strategies were mostly focused on trying to get the person to accept or acknowledge that they had a gambling problem and to a lesser extent reducing access to money and encouraging help seeking. In addition, they identified 12 family-focused strategies that were related to improving well-being (e.g., separation from the person, getting professional help). While reported as family-focused strategies, these were often gambler-focused and involved concealing gambling harm from others and providing financial relief for the gambler. Despite the small sample size, this study indicated that a broad range of approaches may be used by affected others to limit or reduce gambling harm.

To comprehensively understand how affected others respond to gambling harm, different methodological approaches with larger sample sizes are required. One such methodological approach consists of identifying and mapping consumer-derived behaviour change strategies onto well-established behaviour change techniques (BCTs) [37,38]. BCTs reflect the active components of psychotherapeutic interventions in that they are the techniques applied to enact behaviour change. These techniques were first identified through content analysis of treatment manuals and intervention protocols (predominantly physical activity and healthy eating), whereby researchers identified and extracted the range of different techniques administered in behaviour change interventions [39]. Recent research has engaged in a process of top-down and bottom-up approaches involving consensus and data-driven analysis, resulting in a list of 16 broad categories of techniques (e.g., self-monitoring) reflecting some 93 different BCTs [37]. This approach provides a common language to describe the components of behaviour change [37,38] and potentially increases the usability of techniques by having them grouped into meaningful categories [40]. These BCTs can be used as a single technique (e.g., goal setting) or as a combination of techniques (e.g., goal setting, problem solving and action planning) [37]. These BCTs therefore reflect professionally derived and delivered approaches to behaviour change. However, there is very little information on what this means for behaviour change enacted without professional oversight. Research has recently attempted to map consumer-derived behaviour change strategies onto the categories of BCTs for limiting or reducing caffeine and loot box consumption [41,42]. This approach groups consumer-derived behaviour change strategies into higher-order BCT, thereby allowing comparisons between consumer and professionally derived approaches to behaviour change. These studies indicate that consumer-derived behaviour change strategies can be mapped onto higher-level BCTs, but that the nature and nuance of individual strategies differ when enacted without professional guidance. Given that most affected others impacted by gambling harm will never seek treatment, it is imperative that we understand what people naturally do so that these approaches can be better supported.

The aim of the current study was to develop a comprehensive consumer-derived taxonomy that details the broad categories of BCTs used by affected others impacted by gambling harm. This study also aimed to identify and categorize the range of behaviour change strategies that affected others use in real-world settings. Two taxonomies were developed: (i) family-focused and (ii) gambler-focused. Sources for the taxonomies were drawn from internet sources, including consumer-generated content from online gambling message boards and forums internationally and information from websites.

## 2. Methods

### 2.1. Sample and Procedure

A systematic online search was conducted in June 2019 to identify sources (i.e., websites and forums) containing behaviour change techniques used by affected others for the reduction in gambling harm. BCTs were operationalised for plain language as behaviour change strategies, which were defined as actions, both cognitive and behavioural, that were undertaken to limit or reduce gambling harm. We have used this approach previously to translate BCTs into everyday language and systematically represent what consumers do without professional oversight or instruction [41,42]. We use a bottom-up approach to the identification of BCTs by first identifying behaviour change strategies and grouping these into BCTs. This approach is needed so as to represent what consumers naturally do and how they implement these strategies in real-world settings. This study was approved by the University of Auckland Human Participants Ethics Committee (019791).

We used a rigorous search strategy that we developed in earlier research investigating behaviour change strategies for reducing gambling, internet, gaming and pornography, sugar, and caffeine [42,43,44,45]. The search terms included (1) gambling descriptors (i.e., gambling, gambler, slots, fruit machine, pokies, casino); (2) indicators of harm (i.e., harm, stress, loss); (3) target audience (i.e., family, significant other, partner, affected other); and (4) method of change (i.e., strategy, self-help, self-management, cope). We identified six open-access gambling forums and searched these forums using combinations of the search terms describing indicators of harm, target audience, and method of change. The forums were: Gambling Help Online Community Forum (Australia), Gambling Helpline (New Zealand), Gambling Therapy (UK), GamCare (UK), Gamtalk (Canada), and Psych forums (USA). We supplemented this search with a depersonalised search in Google and review of the first five pages of results for each combination of the search terms.

**Figure 1:**
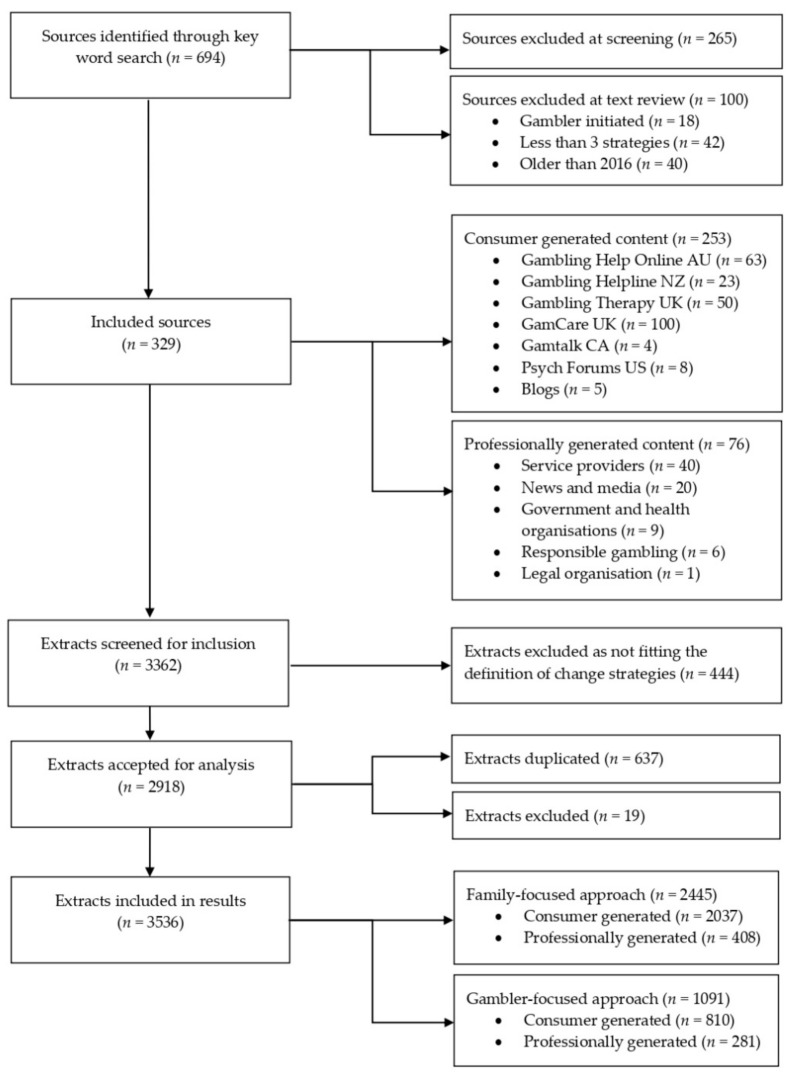
Search process and data extraction.

The search results were reviewed against the following inclusion criteria: (1) focused on gambling and/or gambling harm; (2) were from a family member perspective or were directed towards affected others; (3) included at least three behaviour change strategies; (4) were published since 2016; and (5) were written in the English language. Sources were excluded if they were advertising, promoting or encouraging gambling. The search involving forum threads and comments on websites were limited to the first 20 pages when printed as PDF. This was performed to ensure a representative sample across websites and forums. As indicated in Figure 1, a total of 694 international sources were screened, with 365 excluded because they did not contain behaviour change strategies, were gambler-initiated content or were posted before 2016. Of the 329 included sources, *n* = 253 were consumer generated (e.g., forums, blogs) and 76 professionally generated (e.g., news media, health service providers). The countries of origin were the United Kingdom (*n* = 162, 49.2%), Australia (*n* = 74, 22.5%), the USA (*n* = 51, 15.5%), New Zealand (*n* = 28, 8.5%), Canada (*n* = 9, 2.7%), Ireland (*n* = 3, 0.9%), and Singapore (*n* = 2, 0.6%). Identified sources were archived as PDF and recorded in an Excel database including URLs (Supplementary data 1).

### 2.2. Data Extraction and Preparation

Each PDF of an included source was thoroughly read, and instances of consumer-derived behaviour change strategies were highlighted and copied verbatim into the Excel database. These extracts included the wider context when possible to provide an understanding around the motivation for using the strategy and how the strategy might be implemented. Each extract was linked to the author, origins of the source, title of the source, year posted, and country of origin recorded for traceability. During the extraction, 10% of all PDFs were double checked between research assistants to ensure that all instances containing consumer-derived behaviour change strategies were extracted and those extracted did contain a strategy. The accuracy was between 85% and 100%. The extraction process identified 2918 extracts for analysis.

To prepare the data for coding, each extract was provided a brief descriptor (about 3–5 words) in a separate column capturing an essence of the strategy. As indicated by Newell [46], extracts that contained multiple strategies were duplicated and separated so that each line of text contained a discrete code (*n* = 637 additional extracts identified). Following data cleaning, the original 2918 extracts formed a database of 3536 for analysis.

### 2.3. Development of the Taxonomy

To develop a taxonomy, we analysed the extracted data using a combination of thematic analysis [47,48] and content analysis [46]. The taxonomy was initially developed with the first 500 extracts. These were read for patterns, differences and similarities and emerging themes noted (i.e., strategy category). We iteratively used an inductive and a deductive approach. The deductive approach included behaviour change strategies identified in our previous research [42,43,44,45]. As part of the deductive approach, we cross-checked whether the themes were similar to those found in other behaviour change strategies research in addiction studies. As part of the inductive approach, we allowed new themes to emerge without forcing them into pre-existing themes. A working definition of each theme was developed by NB and SR and used for consistent coding. We used these identified themes to code another 500 extracts and then reviewed the themes. Based on our interpretation of the data, NB and SR made changes to the themes, merging some and splitting others and developed a brief descriptor of each strategy. SR reviewed all matches and disagreements were resolved through discussion. All behaviour change strategies were then mapped onto the BCT categories which provide a common language for understanding the nature of behaviour change [37]. The taxonomy was reviewed by ND, DL and JL and their feedback incorporated.

To increase the usability and clinical relevance of the taxonomy, the identified behaviour change strategies were grouped into the four phases described in the Rubicon model of action phases [42,43,44,45,49]. The Rubicon model holds that all actions fall under four phases: (1) pre-decisional: a phase that includes a range of motivational strategies that help to form an intention to change behaviour; (2) post-decisional: a preparation phase where strategies are focused on planning for the behaviour change; (3) actional phase: a phase which is about enacting actual behaviour change; and (4) post-actional: a phase characterized by a return to motivational strategies, whereby the aim is to decide whether to continue with the behaviour change and if any changes are needed.

### 2.4. Data Coding and Analysis

All analyses were conducted in an Excel database. Data were coded into this taxonomy by NB and two research assistants. SR reviewed and double coded all data (3536 extracts). There were 109 (3.1%) disagreements. All disagreements were resolved between SR and the coders. Results present a summary of the taxonomy with frequency scores calculated for each BCT category. Within each category, we present the behaviour change strategies which reflect consumer-derived approaches to behaviour change. Quotes were kept in their original form except to improve spelling, punctuation and grammar and remove expletives or identifying information.

## 3. Results

### 3.1. Aim 1. Development of the Taxonomy

Two taxonomies of BCTs used by affected others were developed. The first taxonomy encompassed BCTs that aim to minimise, limit or reduce gambling-related harm for affected others (i.e., family-focused), while the second encompassed BCTs that aimed to support a person who gambles to reduce or limit their gambling behaviours (i.e., gambler-focused). The family-focused taxonomy contained 16 BCTs (see Table 1). A total of 2445 statements were coded into the family-focused taxonomy. Family-focused strategies aimed at reducing harm experienced by affected others (e.g., meditate to reduce stress) and, as a rule, did not involve the person who gambled or involved this person only as a receiver of the action (e.g., separate from the person). As indicated in Table 1, the family-focused taxonomy included three pre-decisional techniques (16.6% of family-focused statements), four post-decisional techniques (12.2% of statements), eight actional techniques (70.8% of statements), and one post-actional (0.3% of statements). The most frequent technique was *Professional support* followed by *Financial management* and *Planned consequences*.

The gambler-focused system contained 11 BCTs (see Table 2). A total of 1091 statements involving a gambler-focused approach were coded into the gambler-focused taxonomy. Gambler-focused strategies involved supporting the person who gambles to undertake their own strategies to reduce gambling and/or gambling harm (e.g., family member safeguards the passwords for bank accounts because the person asked them to do so). As indicated in Table 2, the gambler-focused system included two pre-decisional techniques (25.3% of gambler-focused statements), three post-decisional techniques (7.2% of statements), and six actional techniques (67.5% of statements). The most frequent technique was *Feedback on behaviour* followed by *Professional support* and *Financial management*.

Across the two taxonomies, four strategies were not aligned with a specific BCT [37] taxonomy. These included *Communication, Financial management, Maintain momentum,* and *Professional support.* Communication was included in previous taxonomies where it was identified as a skill-based BCT [50]. Financial management relied on a range of other BCTs (such as habit formation, problem solving, and counterconditioning). Maintaining momentum focused on building and maintaining change and also relied on a range of BCTs (such as self-belief, self-talk, and habit formation). *Professional support* was included as a strategy because it was mentioned in terms of seeking specific expert advice or support within the range of other approaches.

### 3.2. Aim 2: Consumer-Derived Behaviour Change Strategies—Family-Focused

#### 3.2.1. Family-Focused: Pre-Decisional

Pre-decisional strategies related to affected others deciding whether they needed to take action on the gambling harm. Decision-making approaches included looking at the pros and cons of whether to be involved, as well as coming to a realisation that there was a problem and seeking knowledge and information. *Pros and cons* focused on the nature of gambling (signs, symptoms, characteristics, and course) as well as the nature of addiction more broadly. Affected others were also interested in getting information on the different options for addressing a problem. This meant that a broad goal had been determined (e.g., repair relationship, address gambling) but the exact action was still to be planned. To inform planning, affected others sought advice from a wide range of internet sources including information on how others had dealt with a gambling problem.

*I’m in a no-win situation. If I stay, he gambles, and the misery continues. If I go, I’m on my own, but at least there is a more promising future.*

*Get informed. If you think your loved one might have a problem, try to learn what you can about gambling addiction, including its warning signs, negative impacts, and options for help and recovery in the community.*

*My wife and I need to build trust. It’s terrible to be in a relationship with no trust. Is there anyone else out there that has some advice on how to rebuild trust?*

*If you look around the forums you will find many other people with similar stories to yourself. Have a talk with a few and see what sorts of things have helped them.*

Affected others reported coming to a realisation that their loved one was experiencing gambling harm. The realisation involved understanding that there was a problem, and that the gambler may be unwilling to admit to a problem or not be ready or willing to change. Affected others also reported coming to realise that they themselves needed help because they had experienced extensive harms or that they themselves needed to make a change. Some affected others discussed this in terms of coming to understand how they had played a role in the gambling problem developing or being maintained.

*I feel like a fool for being so naive and not realising what was happening. I’d just accepted that my husband had changed personality and let him carry on. I really needed help.*

*I was foolish and accompanied her into casinos where she’d play a couple hundred then I’d say time to go. I should have never condoned this because it just allowed her to gamble.*

*All those endless promises that he will change, and not do it again...I now realise they don’t mean anything...I am just a means to an end to him at the moment.*

#### 3.2.2. Family-Focused: Post-Decisional

Post-decisional strategies included setting *Goals and plans*, *Coping planning*, and establishing *Communication* patterns and *Maintaining momentum*. Family goals were mostly focused around setting boundaries as to what is acceptable behaviour from both their own perspective and the gambler’s perspective. *Coping planning* involved the identification of barriers and generation of solutions. These were mostly associated with money-related refusals whereby affected others pre-empted how they would respond to manipulation, threats, bullying, and rationalisations for gambling. There was also a discussion on barriers associated with co-occurring issues (e.g., mental health).

*Set boundaries—know your limits by considering what you are willing to accept and what you will no longer tolerate.*

*Emotionally prepare yourself to deny his requests. For example, remind yourself daily that having money for bills and food is far more important than feeding your husband’s addiction.*

*I suffer from depression, anxiety disorder and OCD—I’ve tried to support him before but now I need to put myself first and I don’t feel like I should have a further burden upon my own mental health.*

Many of the strategies on building *Communication* skills were instructional rather than conveying the person’s own experience of attempting different methods of communication. Communication tended to focus on the logistics, staying calm and remaining non-judgmental. Communication also included the use of threats and ultimatums, which focused mostly on taking strong action such as leaving the relationship or legal action.

*Try and resist the urge to lecture him, but rather express that you’re coming from a place of concern. Gambling can often be a tough topic for people to open up about.*

*I threatened I would go to the police. He broke down and confessed to his parents and mine that he had pawned my jewellery.*

Strategies for *Maintaining momentum* over the longer term were a mix of taking it a day at a time and sticking with goals and plans. Much of the conversation was related to managing expectations about the speed of change and the cycle of behaviour change. It often required family to ‘put on a brave face’ and ‘hang in there’ while remaining ‘on guard and focused’—all at the same time. Strategies also included ways to stay strong and positive, being kind to the self and maintaining hope for improvement.

*Managing addiction is a work in progress. There might come a time when my partner backslides. But if she remains committed to her recovery and treatment, I will remain committed to my vows.*

*But I know it’s a long road ahead. Trying really hard to stay strong…It’s just so hard on top of all of the other life stresses we have going on at the moment. For now, I’ll just keep putting one foot in front of the other.*

*I have a couple of stories with happy endings my friends shared with me, so I just keep them in my mind to strive. Hope our story will end up happily too.*

#### 3.2.3. Family-Focused: Actional

Support approaches included *Professional support* and *Social support* strategies. *Professional support* was the most frequently discussed approach for family. It involved a range of individual and group options. Professional support was sought for initial advice and problem solving and because nothing else was working. For some affected others, professional support was only partially helpful when it did not involve the person with the gambling problem. *Social support* was focused on having someone to confide in and share the burden of the gambling problem. Support was sought from familiar people but also from people on gambling forums. Some affected others reported social support was not always a straightforward option.

*I am exhausted and feel like I am living with a stranger. I have an appointment booked with my Doctor to get plugged into a counsellor to help work through some of my emotions.*

*I went to counselling for myself for a while, but then I realised that it was of no use if we don’t both attend.*

*Don’t forget to look after yourself, you can be there for you partner, support him, but you also need support. Someone you can talk too, confide in, someone who can help you ride the roller coaster, hold your hand when things get tough.*

*It took me a long time to start talking but once I did, I was amazed by how supportive everyone was and still is.*

*I wanted desperately to phone a friend to come get me, but I couldn’t stand the thought of having to explain why I was in tears and everything else.*

One strategy was specifically associated with avoidance from gambling harm. *Avoidance* was focused solely on withdrawal from the gambler either temporarily or permanently. This involved stopping all contact through removing contact details in phones, blocking calls, changing ones phone number and preventing contact with family. Some family talked about being conflicted as to whether avoidance was the best approach, especially when children were involved.

*I have decided that enough is enough. I do love him, but not as a gambler, and I don’t have any energy left for the arguments, lies, deception that comes from this horrible infliction. Time to move on, I guess.*

*I have constantly thoughts about separating from my husband as the only possible way, honestly, I don’t know how it may affect kids’ and our lives either and it scares me.*

Affected others reported engagement in a wide range of activities that directly addressed gambling-related harm. These included *Stress management*, *Financial management*, *Self-monitoring* and *Behavioural substitution*. *Stress management* focused on reducing the psychological, emotional and physical toll of gambling harm. *Behavioural substitution* focused on creating enjoyable habits that improved the affected others life.

*I know I need to look after myself and am doing a mindfulness course at the moment which helps a bit.*

*A healthy diet, regular exercise and adequate rest can improve your wellbeing, and increase your resilience to stress.*

*Take time every day to engage in hobbies, interests, friends, family, things that do not include gambling thoughts of any kind.*

*Financial management* for family included taking control over accounts, budgeting, paying bills and debts, protecting assets, and finding a way to have savings. Affected others were often focused on protecting family money and assets, for example by transferring ownership of property into joint or sole accounts. Affected others also discussed monitoring where they tracked and checked gambling expenditure, frequency and the physical location of the person with the gambling problem.

*I’ve now got every single card and have requested to become power of attorney over his and my mother’s accounts.*

*He had gone into my car, got my wallet and used my card. Luckily, I have learnt to only keep a small amount on my card.*

In addition to monitoring the person with the gambling problem, *Self-monitoring* also involved monitoring of their own feelings to increase awareness of the progress.

*It can help to write down your feelings, even if just to get them off your chest and see how the feelings are changing over time.*

*Planned consequences* included behaviours that were intended to reward or punish the person with the gambling problem in order to shape desired behaviour. This reinforcement was implemented frequently without the gambler’s knowledge, agreement or engagement. The focus was mostly on allowing consequences to occur through the affected other’s absence of action. There was a great deal of discussion on how affected others should examine their own involvement in the gambling problem. Often referred to as ‘enabling’, affected others reflected on how they may have reinforced gambling behaviours through bail outs and preventing natural consequences. In contrast, other discussion was rejecting the concept of ‘enabling’ by affected others and reassuring the family that gambling was not their fault. Rewards for a behaviour that was consistent with family’s goals ranged from praise to paying debt.

*I am letting him make the mistakes he needs to make. At the end of the day, it is his money and me holding it causes a lot of conflict.*

*I wish I could go back in time and never lend you money, never bail you out, never enable the problem. Then maybe you’d hit a smaller rock bottom than we’re now about to hit, maybe a smaller rock bottom back then would be enough for you to want to change.*

*Don’t even entertain that thought one bit. You are not responsible for his gambling. It is all his own doing, his choice.*

*You might consider not lending money if he continues to gamble, however, if he cuts back or stop gambling you might offer to conditionally help to pay off a bill.*

#### 3.2.4. Family-Focused: Post-Actional Phase

*Self-evaluation* was the only strategy in the post-actional phase. This BCT involved assessing and learning from past attempts, deciding future actions, and noticing the difference due to the changed behaviour (e.g., things that improved).

*I’ve tried different strategies on how to help him, but he somehow manages to find his way around everything.*

*I can honestly say I am now thinking about myself. I’ve been on holiday, got my own place, socialise and buy myself things if the notion takes me...things I haven’t done in years and they make me feel good!*

### 3.3. Aim 2: Consumer-Derived Behaviour Change Strategies—Gambler-Focused

#### 3.3.1. Gambler-Focused: Pre-Decisional

Most of the gambler-focused pre-decisional strategies focused on providing *Feedback* on the impact of behaviour or *Providing information*. *Feedback* focused on the impact of the gambling on affected others including psychological, emotional and physical health, relationship and children, finances, and fear of the future. Feedback was offered to encourage the person to think about their gambling and whether they have a problem. The way feedback was offered varied from gently probing through to questions and reflection to forcefully telling the person what they had done or become.

*I try to ask questions to make him think about his gambling and its effect. I can’t say it’s actually working but he is at least talking every now and again and sometimes I can see something I have asked ticking in his head.*

*Tell the person how his or her gambling affects you and the family—Partners of gamblers often feel confused, angry and helpless. Don’t be afraid to tell them how their gambling affects you but remain calm.*

*We went to the Bank and printed out all the statements and highlighted the amounts that had been spent on gambling. It came to $47,000.*

Affected others also provided the gambler with information. This information addressed issues such as the symptoms of gambling problems, how gambling works, how to know if there is a problem, and treatment options. Some family said that they asked the person what information would be helpful or offered it directly. Others commented on leaving the information in a discrete place that could be found by the gambler.

*Download the GA 20-questions and show them to him—maybe he doesn’t realise his problem is a recognised addiction and that there is support for him.*

*Ask him what he thinks would be helpful and offer to support him by getting more information or seeking external assistance.*

*I leave gambling pamphlets where he can see them in the hope he may pick it up when no-one is looking.*

#### 3.3.2. Gambler-Focused: Post-Decisional

The post-decisional phase included establishing *Goals and plans*, developing *Coping plans* and *Maintaining momentum*. We also identified *Communication* as relevant to gamblers in this phase but were unable to differentiate it from family-focused approaches (this BCT is therefore only reported in the family-focused section). Establishing *Goals and plans* was focused on supporting the person to establish a plan that was agreeable to both the gambler and affected others. Many goals and plans that were gambler-focused indicated that affected others may be making plans *for* the gambler rather than *with* the gambler.

*Make an action plan if you are helping someone close to you recover from problem gambling, it can be useful to have a plan in place to help keep them headed in the right direction.*

*Work with the person to agree on acceptable behaviours, e.g., talking to a professional, staying within agreed spending limits.*

*I am setting little goals (like deleting online accounts) with dates for it to be done by to make it more manageable. Are there any other suggestions I can to do to make this easier for him?*

*Coping plans* were focused predominantly on the identification of triggers and preparedness for urges and cravings to prevent slips and relapses. Affected others were focused on the reality that the person with the gambling problem was likely to return to unwanted gambling at some point. Although some of the plans included practical help for controlling urges, most were fairly rudimentary in terms of offering support and not being judgmental. Moreover, there was little evidence of identifying a specific response to lapses and relapses. Affected others also identified multiple co-occurring or underlying issues related to the gambler that were barriers to reaching goals and enacting plans. These including mental health issues such as depression, anxiety, grief and loss.

*Help the person identify their triggers. This can help them know what to avoid or allow them to learn how to cope when faced with these triggers.*

*Let him know that he can call you when he’s having an urge to gamble—sometimes that can be really powerful, as it helps the gambler realise that the urge will pass by talking to someone or doing something to distract themselves.*

*Don’t get angry or frustrated with them if they slip from time to time—this is normal and you can encourage them to learn from these mistakes.*

*We identified the underlying issues that have triggered this—we lost a baby and his mum, and he hasn’t dealt with this.*

*Maintaining momentum* involved helping the person with the gambling problem stick to their plans and goals. Affected others reported providing encouragement and celebrating gambling-related milestones and achievements.

*I’m also happy to share that yesterday was his two-week anniversary of giving up gambling. He’s having trouble celebrating his anniversaries so far I think so I’m trying to hold the hope for him until he feels it himself.*

#### 3.3.3. Gambler-Focused Approach: Actional Phase

Actional strategies focused on methods to support the gambler in taking action. The most frequent strategies were related to *Finances and cash control* and *Social* and *Professional support*. For *Professional support*, affected others provided information on treatments and referrals, offered direct support for making or attending an appointment and also offered to attend alongside the gambler.

*I went to counselling sessions with my husband a couple of times and I believe it helped both of us to understand each other a bit better.*

*When I feel the time is right again, I’ll suggest he calls the helpline. I think talking helps my dad immensely. He’s probably just terrified to do it in a public forum.*

*Social support* referred to the types of roles family can play as well as how they can deliver support (e.g., non-judgmentally). The types of roles ranged from being generally supportive to helping during crisis. Support was not always straightforward with affected others questioning how much support they could offer, especially in crisis situations such as suicidal ideation. It was unclear whether social support was always requested by the gambler or whether affected others were offering a type of support that they thought was best suited for the situation.

*My mum still has a few issues to work out and we will always be here to help her. We are slowly working out how best to do that as a family.*

*I got an awful text from my boyfriend who is an addict and has been for a long time. He was saying goodbye. That he needed to be free and this (suicide) was the only way. Thank god I managed to get him to come home and talked to him. I think I managed to save him today. And I’m terrified about tomorrow and the days to come.*

*Behavioural substitution* was a related approach whereby family could support refocusing away from gambling and towards alternative hobbies and interests.

*Fortunately, Sue has a real talent with eBay. I’ve got a business friend of mine who wants her to create a shop there. This will keep Sue busy with other stuff thereby cutting down on the spare time she has to play the pokies.*

Affected others were very active in supporting *Finances and cash control*. These strategies included supporting the person to budget, pay bills, manage debt, and organise everyday banking to minimise gambling-related harm. Strategies also included minimising the amount of available cash.

*I have helped her create and stick to a budget, rang and arranged payment plans with finance companies, tracked her spending via internet banking, and held on to her ATM card.*

*Discuss with the person how they can limit their access to cash or credit. This will remove a major gambling trigger.*

Although there was a notion that gambler-focused financial management was performed on the gambler’s request and/or together with the person, some actions were done *to* the gambler.

*I took care of all expenditure of the house including food, bills, house, everything, I also gave her a bit of money every month to make her feel life is still happy. But the situation was worsening since she kept asking for more money.*

Similar to some financial control strategies, the *Avoidance* strategy also involved affected others doing things *to* the gambler in an attempt to be helpful. Some affected others were, however, clearly working with the gambler to implement an avoidance strategy such as removing apps or installing a gambling blocker.

*He is terrible with his spending and will also gamble online. I have deleted the gambling apps off his phone and said I never want to see them on your phone again.*

*I would pretend I was him and self-exclude him from the sites he played.*

*Sit with him and make a list of all online gambling sites and self-exclude. Then do the same with all bookmakers he could possibly go to.*

*Consumption planning* was the least frequently discussed strategy. Consumption planning was specifically about accepting that gambling will continue and supporting gamblers to control their expenditure. Affected others were involved in setting agreed spending limits.

*Part of me thinks I should come to an agreement with her on how often and how much she can spend per week. So that she can be happy, and I can learn to deal with the occasional gamble.*

*We figured into our budget $50.00 a week each, which we may do whatever we like with. I don’t mind if he wishes to gamble that $50.00 a week—it is his discretionary spending and he is free to decide how to spend it.*

## 4. Discussion

The aim of this study was to identify the BCTs and associated behaviour change strategies used by affected others to limit or reduce the impact of gambling harm. We investigated real-life stories from affected others self-reported on gambling forums and websites and various advice provided to affected others online. We developed two taxonomies which were organised into the Rubicon model of action phases: a family-focused taxonomy (16 BCTs) and a gambler-focused taxonomy (11 BCTs). Over 3000 statements were coded into these two systems representing the views of affected others all over the world.

Lived experience data broadly aligned with BCTs that are used in treatment for families impacted by gambling. In particular, we found that affected others engage in activities that are similar to CRAFT interventions [27,30] in which they attempt to reinforce desirable behaviours. Similarly, the emphasis on support and stress management apparent in the SSCS model [17] was also frequently reported in the current sample. Across both the family-focused and gambler-focused taxonomies, there was a strong focus on prompting help seeking by the gambler and/or affected others, which is also consistent with the CRAFT and SSCS models. Goals and planning did not feature substantially in approaches used by lay people. However, feedback and monitoring were prominent, especially in the gambler-focused approach.

This is the first comprehensive international account examining consumer-derived behaviour change strategies which reflect what consumers do in real-world settings. Many of the behaviour change strategies implemented by affected others were consistent with our other studies involving a range of other addictions [41,42,43,44,45]. Mapping the strategies into the phases of the Rubicon model [49] helped with distinguishing actions taken at the start of behaviour change to become motivated to change (i.e., Is there a problem? Do I need to do something?), during planning (i.e., what, when and how to do), actions that carry out behaviour change (e.g., cancel joint accounts), and actions taken at the end of behaviour change (i.e., to assess this attempt and decide what to do next). It also showed the potential gaps in unassisted use of the behaviour change strategies and areas where implementation was challenging. Similar to gamblers who experience implementation failure [51], affected others also reported strategies not working as intended or being counterproductive. This might be related to the finding that a very small number of strategies were dedicated to goal setting and planning in the post-decisional phase (5.2% in family-focused and 5% in gambler-focused data). This low frequency of goals and planning is similar to our previous findings for people with problems associated with internet, gaming, pornography, sugar, and caffeine [42,44,45]. Future interventions based on self-directed use of behaviour change strategies may benefit from a stronger focus on goal setting and planning element.

This is also the first comprehensive account of gambler-focused approaches to behaviour change. Gambler-focused approaches were intended to support the person with the gambling problem. Across the data, there was an indication that some affected others asked gamblers how they could help and offered their skills, time or expertise in problem solving, setting plans and implementing strategies. However, a strong pattern in the gambler-focused data was that affected others often acted without invitation or discussion with the gambler. This is not unexpected, given that affected others carry the burden of harm and experience high levels of stress and strain. Help for affected others who select a gambler-focused approach is available. For example, Mental Health First Aid Guidelines for gambling problems [26] and CRAFT interventions [27,29,30,33] provide information on the signs of a problem, how to raise the issue and methods and options for referral. However, these guidelines and interventions are not intended to provide a wide range of specific information on how a family member might support a person with a gambling problem, hence the need for the development of further, more specific resources.

Some professionally delivered interventions offer a blended approach that is both family- and gambler-focused [27,29,30,33]. In our study, affected others were conflicted as to the best course of action once a problem was identified. This conflict was related to balancing their needs with those of the person experiencing the gambling problem. Future research should examine who would most benefit from a family-focused, gambler-focused or blended approach. We reported communication as a family-focused approach, but it clearly applied to both family- and gambler-focused actions. We were unable to identify the purpose or intent of different communication styles and as such kept the data as a single category. Most of the communication techniques discussed were around ways to talk about the gambling problem with very little information on trouble shooting (e.g., what to do when the person does not want to talk or where there is conflict). In intervention development, we recommend that communication is included in all interventions involving family as it underpins many of the identified techniques and strategies.

### 4.1. Limitations

This study is the first systematic attempt to classify strategies used by affected others for gambling harm reduction, but it is not without limitations. First, data were sourced from online forums which reflects a particular subgroup of affected others. It may be that affected others who post online did not have success with managing gambling harm or affected others who experience less severe problems do not post in these forums. To mitigate this, we extracted a very large volume of data (over 3500 extracts) from 329 sources. Second, international data identification and extraction was both a strength and a weakness of this study. It may be that some strategies that are specific only to certain contexts may have been omitted or minimised by merging such a large and culturally diverse dataset. Future research should consider validating the included strategies in specific regions or cultures. Third, the behaviour change strategies outlined in this document represent a range of opinions and experiences derived largely from lived experience but the current methodology does not allow us to determine the effectiveness of these strategies or the effectiveness of their implementation. To address this in part, we mapped consumer-derived behaviour change strategies onto the well-established and evidence-based BCT framework.

While our focus in this study was not on effectiveness or implementation success, quotes indicated some behaviour change strategies might be more helpful. For example, financial management was probably the most poorly implemented strategy. We found evidence that conflict was associated with haphazard implementation which resulted in further conflict within the family unit. For instance, some affected others found out about the gambling and reacted by immediately cutting the gambler off from all cash and taking control over all family finances. With time, affected others gave some or full control back to the gambler because of the pressure from the gambler, mental exhaustion, thinking the problem was resolved or feeling over-burdened. Other research indicates that the financial harms from gambling are enduring and can lead to generations of debt transfer [4,5,6]. CRAFT interventions [27,29,30,33] offer some financial management strategies. However, they lack specific details and the range of options on how to have the conversation, how to select an approach and how to implement it over the longer term. To address this issue, future research should examine implementation issues associated with behaviour change strategies for affected others and their impact on strategy effectiveness. This information can then be subject to further evaluation to determine which strategies work for whom and in which situations.

### 4.2. Clinical Implications

Research suggests that gamblers combine behaviour change strategies to form a self-directed approach [52]. Multiple studies indicate that a combination of up to ten or more strategies is often needed to mitigate all the different scenarios that arise when changing behaviour (e.g., avoid gambling, get busy, reduce access to cash, increase support) [52]. The current study indicates family may follow a similar approach, whereby a combination of strategies is helpful. Future research should examine the optimal number of strategies and whether this changes over time in response to changing need.

There is an urgent need for an increased number of resources to support family. First, we identified a preference for family to leave information around the house so as to prompt gambler engagement. Resources could be developed whereby affected others complete part of a booklet involving feedback to the gambler with the remainder prompting input from the gambler. The current study identified specific information that could be part of these resources from both a family- and gambler-focused approach. Second, we found very little evidence that affected others knew how to support a person experiencing gambling cravings or urges or assist them to refocus away from gambling. Resources could be developed that offer peer-to-peer (including family) interventions, whereby affected others are trained to provide support according to what is helpful for the gambler. Similarly, comparisons with BCTs can be made to identify gaps or weaknesses in consumer-derived approaches. For instance, the current study found that affected others focused on monitoring the gambler (but not their own behaviour) and did not seek to support the gambler to self-monitor. Including peer support in self-monitoring apps and interventions may help to involve affected others who wants to support gambling change.

The BCTs and strategies outlined in this document can form bottom-up interventions that are grounded in lived experience. This approach means intervention content does not need to be subject to knowledge translation as the content would already be in lay language and reflect what people naturally do. Ideally, the intervention follows the natural course and allows individuals to self-select their own tailored and personalised intervention. The effectiveness of this approach needs to be determined including the identification of strategies that should not be used in certain situations (e.g., managing finances when living with domestic violence). These findings can then guide the development of self-assessment tools to guide personalisation. Similarly, consumption planning associated with a goal of reduced gambling may not be practical or feasible where family have been severely harmed financially. We also found that some strategies can be dual focus. For example, under cash control and financial management, affected others can set their own budget and bills payments (family-focused), or they can help the person who gambles to set a budget and bills payments (gambler-focused). In the case of joint households, the differentiation was subtle, which shows that, from a practical perspective, there might be a need to encourage a blended approach. This need for a blended approach was noted in other research investigating the treatment preferences of affected others of people who gamble.

## Figures and Tables

**Table 1:** Family-focused taxonomy and results for consumer-derived BCTs.

BCT Category	Description of Consumer-Derived BCT	Number and %	
***Pre-decisional phase***			
Pros and cons	Weigh the available evidence for and against taking action. Pros and cons were focused predominantly on the degree of involvement in the gambling problem and whether to initiate, maintain or avoid engagement.	148	6.1%
Realisation	Come to realise, accept or acknowledge the development or presence of gambling harm. Realise the extent of harm and burden on the family. Realise that an action may be required.	126	5.2%
Seek knowledge and information	Understand the nature of gambling problems and addiction and possible harm to the family. Information was sought from a wide range of sources including peer-to-peer forums, books and gambling harm reduction websites.	133	5.4%
***Post-decisional phase***			
Goals and plans	Set priorities and goals that are focused on the self or children and other affected others. Establish expectations and convey boundaries for own behaviour on gambling-related issues (e.g., moral but not financial support).	75	3.1%
Communication	Establish communication patterns that involve being prepared, enhanced listening skills and methods to communicate own needs. Strategies include communication that is viewed by consumers as unhelpful (accusations, threats, ultimatums, nagging, lecturing or prompting guilt or blame) and helpful (focus on gambling behaviours as being unacceptable rather than the person).	137	5.6%
Coping planning	Plan for barriers that might get in the way of behaviour change. Barriers were predominately related to pressure from a gambler requesting money or family member’s pre-existing conditions such as depression or anxiety.	53	2.2%
Maintain momentum	Maintain family member’s change in response to general barriers such as shifting readiness, importance and priorities. Families do this through practicing patience, willpower, determination and renewing commitment.	34	1.4%
***Actional phase***			
Avoidance	Distance from the person with the gambling problem. Distance may be physical or psychological. It may involve temporary or permanent separation. Prepare for distancing by establishing financial independence and organising social support, housing and a safety plan.	160	6.5%
Behavioural substitution	Refocus away from gambling harm and towards new or improved habits. For family, this included habits that are enjoyable, good for personal development, and can be performed jointly or separately.	7	0.3%
Financial management	Act to investigate, audit, assess and address financial harm. Arrange for the repayment of debt and ensure processes in place to stem the flow of cash for gambling. Install safeguards to protect current and future assets.	334	13.7%
Planned consequences	Reinforce desired behaviour of the gambler through selective rewards and exposure to negative consequences. Look at role of affected others in current and past reinforcement (e.g., enabling behaviours).	289	11.8%
Professional support	Seek support, advice or treatment from professionals or treatment services. This includes seeking expertise from peer support groups and in psychology, psychiatry, medicine, finances, law, housing, and family violence. Reasons are to learn new skills, build confidence and increase support network.	547	22.4%
Social support	Identify the different types of social support such as practical or emotional. Develop a willingness and skills to ask and receive support. Be a support person for others, as well as share information and updates with family. Get and give inspiration to other people.	117	4.8%
Stress management	Identify the signs of stress and how stress can make it difficult to respond to gambling harm. Enact strategies to manage stress including self-care, self-talk, and relaxation. Improve resilience through healthy diet, exercise and adequate sleep. Learn to notice and name emotions and respond in a way that is helpful.	138	5.6%
Self-monitoring	Establish the focus and methods to monitor behaviours against goals and plans. Monitoring was mostly focused on tracking the gambler’s behaviours against the goals of affected others. This involved regular checking of banking transactions, physical location and questioning the person on their movements. Monitoring was also used to increase gambler’s accountability.	140	5.7%
***Post-actional phase***			
Self-evaluation	Assess attempts of behaviour change to learn from them and decide what to do next. Notice the difference the change in the behaviour have created (e.g., what has improved).	7	0.3%
Total		2445	100%

**Table 2:** Gambler-focused taxonomy and results for consumer-derived BCTs.

BCT Category	Description of Consumer-Derived BCT	Number and %	
***Pre-decisional phase***			
Feedback on behaviour	Provide evidence (e.g., bank statements) on how gambling behaviours are causing harm to the family with the intention of prompting increased awareness. Harms can include finances as well as relational, emotional, and physical health. Prompt gambler to take a test to assess their gambling.	250	22.9%
Provide information	Provide information on gambling problems and how gambling works. Provide the information in a respectful and direct way, e.g., make it available for the person without pressuring them to read it.	26	2.4%
***Post-decisional phase***			
Coping planning	Help the gambler to plan for lapses and relapses and to manage barriers to change. This focused on identifying and responding to gambling triggers, cravings and urges in a goal-consistent manner. Triggers included underlying causes and/or co-occurring substance use or mental health concerns. Help included practical support such as being a distraction in the moment of an urge.	39	3.6%
Goals and plans	Support gambler’s goal setting and planning by offering to jointly construct or discuss the content of goals and plans. Encourage those involved to agree to the outcome of discussion.	16	1.5%
Maintain momentum	Support gambler to maintain behaviour change in response to general barriers such as shifting priorities and readiness to change. Understand the nature and pace of behaviour change to support the current level of readiness. Provide encouragement and praise, recognise and celebrate gambler’s achievements.	24	2.2%
***Actional phase***			
Avoidance	Support the person to avoid gambling or gambling triggers through discussion on different options (e.g., self-exclusion). Offer to help with self-exclusion, change passwords, remove betting apps or install blocking software.	77	7.1%
Behavioural substitution	Support the gambler to develop interests and hobbies away from gambling. Assist the person to refocus their attention towards family or life without gambling.	19	1.7%
Consumption planning	Support the person to reach a goal of gambling reduction through a focus on preparation for a gambling episode. This includes setting a limit for the frequency, time, and amount of expenditure. It also includes enacting strategies during and post-gambling for sticking to these limits.	12	1.1%
Finances and cash control	Offer advice, assistance or information on options for financial management and budgeting. This includes debt and bill management. Agree on a method to manage access to cash that may involve affected others offering to oversee the finances for a period of time.	208	19.1%
Professional support	Encourage and support treatment seeking. This may involve getting information, offering support to make an appointment, attending an appointment or offering post-appointment support. Professional support includes psychological, psychiatric, medical, or financial advice. Support can involve individual or group, and online, telephone or face-to-face support.	240	22.0%
Social support	Families can provide support to the person who gambles and help them to get more support from others. Support can be about helping the gambler to open up about their gambling or during a crisis.	180	16.5%
Total		1091	100%

## Supplementary Materials

The following are available online at https://www.mdpi.com/2077-0383/10/4/583/s1, Supplementary data 1: Affected other sources and extracts.

## References

[1] American Psychiatric Association (2013). Diagnostic and Statistical Manual of Mental Disorders (DSM-5).

[2] Di Nicola M., De Crescenzo F., D’Alò G.L., Remondi C., Panaccione I., Moccia L., Molinaro M., Dattoli L., Lauriola A., Martinelli S. (2020). Pharmacological and psychosocial treatment of adults with gambling disorder: A meta-review. J. Addict. Med..

[3] Langham E., Thorne H., Browne M., Donaldson P., Rose J., Rockloff M. (2016). Understanding gambling related harm: A proposed definition, conceptual framework, and taxonomy of harms. BMC Public Health.

[4] Kourgiantakis T., Saint-Jacques M.-C., Tremblay J. (2013). Problem gambling and families: A systematic review. J. Soc. Work Pract. Addict..

[5] Riley B.J., Harvey P., Crisp B.R., Battersby M., Lawn S. (2018). Gambling-Related harm as reported by concerned significant others: A systematic review and meta-synthesis of empirical studies. J. Fam. Stud..

[6] Browne M., Bellringer M., Greer N., Kolandai-Matchett K., Langham E., Rockloff M., Du Preez K., Abbott M. (2017). Measuring the Burden of Gambling Harm in New Zealand.

[7] Goodwin B.C., Browne M., Rockloff M., Rose J. (2017). A typical problem gambler affects six others. Int. Gambl. Stud..

[8] Rockloff M.J., Browne M., Russell A.M.T., Merkouris S.S., Dowling N.A. (2019). A quantification of the net consumer surplus from gambling participation. J. Gambl. Stud..

[9] Salonen A.H., Alho H., Castrén S. (2015). Gambling frequency, gambling problems and concerned significant others of problem gamblers in Finland: Cross-sectional population studies in 2007 and 2011. Scand. J. Public Health.

[10] Salonen A.H., Alho H., Castren S. (2016). The extent and type of gambling harms for concerned significant others: A cross-sectional population study in Finland. Scand. J. Public Health.

[11] Salonen A.H., Castrén S., Alho H., Lahti T. (2014). Concerned significant others of people with gambling problems in Finland: A cross-sectional population study. BMC Public Health.

[12] Shiue I. (2015). Self and environmental exposures to drinking, smoking, gambling or video game addiction are associated with adult hypertension, heart and cerebrovascular diseases, allergy, self-rated health and happiness: Japanese General Social Survey, 2010. Int. J. Cardiol..

[13] Svensson J., Romild U., Shepherdson E. (2013). The concerned significant others of people with gambling problems in a national representative sample in Sweden—A 1 year follow-up study. BMC Public Health.

[14] Wenzel H.G., Øren A., Bakken I.J. (2008). Gambling problems in the family—A stratified probability sample study of prevalence and reported consequences. BMC Public Health.

[15] Dowling N.A., Rodda S.N., Lubman D.I., Jackson A.C. (2014). The impacts of problem gambling on concerned significant others accessing web-based counselling. Addict. Behav..

[16] Rodda S.N., Dowling N.A., Thomas A.C., Bagot K.L., Lubman D.I. (2019). Treatment for Family Members of People Experiencing Gambling Problems: Family Members Want Both Gambler-Focused and Family-Focused Options. Int. J. Ment. Health Addict..

[17] Orford J., Copello A., Velleman R., Templeton L. (2010). Family members affected by a close relative’s addiction: The stress-strain-coping-support model. Drugs Educ. Prev. Policy.

[18] Copello A., Templeton L., Orford J., Velleman R. (2010). The 5-Step Method: Principles and practice. Drugs Educ. Prev. Policy.

[19] Orford J., Cousins J., Smith N., Bowden-Jones H. (2017). Stress, strain, coping and social support for affected family members attending the National Problem Gambling Clinic, London. Int. Gambl. Stud..

[20] Rychtarik R.G., McGillicuddy N.B. (2006). Preliminary evaluation of a coping skills training program for those with a pathological-gambling partner. J. Gambl. Stud..

[21] Hobfoll S.E., Spielberger C.D. (1992). Family stress: Integrating theory and measurement. J. Fam. Psychol..

[22] Moos R.H., Finney J.W., Cronkite R.C. (1990). Alcoholism Treatment: Context, Process, and Outcome.

[23] Rychtarik R.G., McGillicuddy N.B. (2005). Coping skills training and 12-step facilitation for women whose partner has alcoholism: Effects on depression, the partner’s drinking, and partner physical violence. J. Consult. Clin. Psychol..

[24] Buchner U.G., Koytek A., Wodarz N., Wolstein J. (2019). Is an e-mental health programme a viable way to reach affected others of disordered gamblers? A feasibility study focusing on access and retention. Int. Gambl. Stud..

[25] Côté M., Tremblay J., Jiménez-Murcia S., Fernàndez-Aranda F., Brunelle N. (2019). How Can Partners Influence the Gambling Habits of Their Gambler Spouse?. J. Gambl. Stud..

[26] Bond K.S., Jorm A.F., Miller H.E., Rodda S.N., Reavley N.J., Kelly C.M., Kitchener B.A. (2016). How a concerned family member, friend or member of the public can help someone with gambling problems: A Delphi consensus study. BMC Psychol..

[27] Hodgins D.C., Toneatto T., Makarchuk K., Skinner W., Vincent S. (2007). Minimal treatment approaches for concerned significant others of problem gamblers: A randomized controlled trial. J. Gambl. Stud..

[28] Magnusson K., Nilsson A., Gumpert C.H., Andersson G., Carlbring P. (2015). Internet-Delivered cognitive-behavioural therapy for concerned significant others of people with problem gambling: Study protocol for a randomised wait-list controlled trial. BMJ Open.

[29] Magnusson K., Nilsson A., Andersson G., Hellner C., Carlbring P. (2019). Internet-Delivered cognitive-behavioral therapy for significant others of treatment-refusing problem gamblers: A randomized wait-list controlled trial. J. Consult. Clin. Psychol..

[30] Nayoski N., Hodgins D.C. (2016). The efficacy of individual community reinforcement and family training (CRAFT) for concerned significant others of problem gamblers. J. Gambl. Issues.

[31] Hing N., Tiyce M., Holdsworth L., Nuske E. (2013). All in the family: Help-Seeking by significant others of problem gamblers. Int. J. Ment. Health Addict..

[32] Archer M., Harwood H., Stevelink S., Rafferty L., Greenberg N. (2019). Community reinforcement and family training and rates of treatment entry: A systematic review. Addiction.

[33] Makarchuk K., Hodgins D.C., Peden N. (2002). Development of a brief intervention for concerned significant others of problem gamblers. Addict. Disord. Treat..

[34] Merkouris S.S., Dowling N.A., Rodda S.N. (2020). Affected Other Treatments: Systematic Review and Meta-Analysis Across Addictions.

[35] Rodda S.N., Lubman D., Dowling N., McCann T. (2013). Reasons for using web-based counselling among family and friends impacted by problem gambling. Asian J. Gambl. Issues Public Health.

[36] Côté M., Tremblay J., Brunelle N. (2018). A new look at the coping strategies used by the partners of pathological gamblers. J. Gambl. Issues.

[37] Michie S., Richardson M., Johnston M., Abraham C., Francis J., Hardeman W., Eccles M.P., Cane J., Wood C.E. (2013). The behavior change technique taxonomy (v1) of 93 hierarchically clustered techniques: Building an international consensus for the reporting of behavior change interventions. Ann. Behav. Med..

[38] Michie S., West R., Sheals K., Godinho C.A. (2018). Evaluating the effectiveness of behavior change techniques in health-related behavior: A scoping review of methods used. Transl. Behav. Med..

[39] Abraham C., Michie S. (2008). A taxonomy of behavior change techniques used in interventions. Health Psychol..

[40] Cane J., Richardson M., Johnston M., Ladha R., Michie S. (2015). From lists of behaviour change techniques (BCT s) to structured hierarchies: Comparison of two methods of developing a hierarchy of BCT s. Br. J. Health Psychol..

[41] Gong L., Rodda S.N. (2020). An Exploratory Study of Individual and Parental Techniques for Limiting Loot Box Consumption. Int. J. Ment. Health Addict..

[42] Rodda S.N., Booth N., McKean J., Chung A., Park J., Ware P. (2020). Mechanisms for the reduction of caffeine consumption: What, how and why. Drug Alcohol Depend..

[43] Rodda S.N., Hing N., Hodgins D.C., Cheetham A., Dickins M., Lubman D.I. (2018). Behaviour change strategies for problem gambling: An analysis of online posts. Int. Gambl. Stud..

[44] Rodda S.N., Booth N., Brittain M., McKean J., Thornley S. (2020). I was truly addicted to sugar: A consumer-focused classification system of behaviour change strategies for sugar reduction. Appetite.

[45] Rodda S.N., Booth N., Vacaru M., Knaebe B., Hodgins D. (2018). Behaviour change strategies for internet, pornography and gaming addiction: A taxonomy and content analysis of professional and consumer websites. Comput. Hum. Behav..

[46] Newell R. (2011). Research for Evidence-Based Practice in Healthcare.

[47] Braun V., Clarke V. (2006). Using thematic analysis in psychology. Qual. Res. Psychol..

[48] Braun V., Clarke V., Hayfield N., Terry G., Liamputtong P. (2019). Thematic analysis. Handbook of Research Methods in Health Social Sciences.

[49] Heckhausen H., Gollwitzer P.M. (1987). Thought contents and cognitive functioning in motivational versus volitional states of mind. Motiv. Emot..

[50] Michie S., Whittington C., Hamoudi Z., Zarnani F., Tober G., West R. (2012). Identification of behaviour change techniques to reduce excessive alcohol consumption. Addiction.

[51] Rodda S.N., Hing N., Hodgins D.C., Cheetham A., Dickins M., Lubman D.I. (2017). Change strategies and associated implementation challenges: An analysis of online counselling sessions. J. Gambl. Stud..

[52] Rodda S.N., Bagot K., Cheetham A., Hodgins D.C., Hing N., Lubman D.I. (2018). Types of change strategies for limiting or reducing gambling behaviours and their perceived helpfulness: A factor analysis. Psychol. Addict. Behav..

